# Genetically controlling VACUOLAR PHOSPHATE TRANSPORTER 1 contributes to low-phosphorus seeds in Arabidopsis

**DOI:** 10.1080/15592324.2023.2186641

**Published:** 2023-03-08

**Authors:** Guangfang Sun, Mingda Luan, Jiansheng Wen, Bin Wang, Wenzhi Lan

**Affiliations:** a School of Life Sciences, Nanjing University, Nanjing, Jiangsu, China; b Institute of Future Agriculture, Northwest Agriculture and Forestry University, Yangling, Shaanxi, China

**Keywords:** VPT1, Pi distribution pattern, long-distance Pi transport, floral organ, low-P seeds

## Abstract

Phosphorus (P) is an indispensable nutrient for seed germination, but the seeds always store excessive P over demand. High-P seeds of feeding crops lead to environmental and nutrition issues, because phytic acid (PA), the major form of P in seeds, cannot be digested by mono-gastric animals. Therefore, reduction of P level in seeds has become an imperative task in agriculture. Our study here suggested that both *VPT1* and *VPT3*, two vacuolar phosphate transporters responsible for vacuolar Pi sequestration, were downregulated in leaves during the flowering stage, which led to less Pi accumulated in leaves and more Pi allocated to reproductive organs, and thus high-P containing seeds. To reduce the total P content in seeds, we genetically regulated VPT1 during the flowering stage and found that overexpression of VPT1 in leaves could reduce P content in seeds without affecting the production and seed vigor. Therefore, our finding provides a potential strategy to reduce the P level of the seeds to prevent the nutrition over-accumulation pollution.

## Introduction

Phosphorus (P) is an essential macronutrient for all bio-organisms, while phosphate (Pi) is the major form of P that can be utilized by plants. Because of its pivotal roles in numerous primary bio-metabolisms, such as formation of nucleic acids, lipid synthesis, protein phosphorylation, etc., Pi is indispensable for plant growth and development. Pi transporters are known as the functional components that facilitate Pi transportation and contribute to fine-tuning of Pi homeostasis in plants^1,2^. So far, vacuole is defined as the biggest Pi pool that is critical for maintaining Pi balance in plant cells. People have made big progresses in identification of the long-sought vacuolar Pi transporters, recently. VPT1 (Vacuolar Phosphate Transporter 1, also named PHT5;1) family members were found to be the vacuolar Pi influx transporters that are responsible for vacuolar Pi storage under Pi sufficient conditions^3,4^, while VPE (Vacuolar Phosphate Efflux) family members were identified as the vacuolar Pi efflux transporters that contribute to vacuolar Pi remobilization in response to Pi deficiency^5^. These two types of vacuolar transporters are vital for Pi homeostasis in plants.

With the assistance of multiple transporters, Pi is transported into or out-of various organelles, cells and tissues for acclimating to the physiological demand of plants. Especially, in different development stages, the plant adopts different policies for Pi distribution in tissues. During the flowering stage, physiological changes and expression of specific genes would cater to reproduction. Pi is essential for reproduction, and sufficient available Pi contributes to the transformation from vegetative growth to flowering in plants. Additionally, from flowering to seed maturation, large amounts of Pi are allocated to the reproductive organs for synthesis of phytic acid (PA, or InsP_6_) which is referred as the major stored form of P in seeds^6–8^. Although PA is required for germination of seeds, most crop seeds contain much higher PA beyond the quantity of demand^9^. As many crop seeds such as corn and sorghum seeds are often used for animal feed, this high level of PA in seeds leads to severe water eutrophication. It has been known that mono-gastric animals cannot digest PA and most of PA in feed is excreted into lakes, rivers and the groundwater^10^. Moreover, PA could easily chelate Fe, Cu and Zn that are essential elements for the activities of digestive enzymes; thus, high level of PA contributes to malnutrition of the animals^10^. Therefore, PA content or P accumulation in seeds should be reduced to solve these environmental and nutritional issues. However, suppressing the allocation of P to the seeds by using efficient genetic target is largely unexplored.

Our previous work suggested that VPT1 family members were essential for maintaining the systemic Pi balance during flowering. Loss of function of VPT1 and VPT3 would affect the long-distance Pi transport and result in toxic level of Pi allocated to the floral organ rather than stored in vacuole of leaves^11^. Therefore, genetically controlling the VPT proteins may reduce the Pi level in reproductive organs and seeds. In this study, we uncovered that the expression of *VPT1* and *VPT3* was downregulated in leaves during flowering stage; this regulation may contribute to more Pi allocated to reproductive organs for enhancement of the PA synthesis in seeds. Through a dexamethasone-inducible expression system, we found that overexpression of VPT1 in leaves resulted in less Pi in the stem xylem sap and thus reduced P accumulation in the floral organ and seeds. Our finding should provide available genetic tools for generating the low-P grain crops.

## Results and discussion

### Vacuolar Pi transporter genes are transcriptionally regulated for controlling Pi status in the reproductive organ

**Figure 1.**
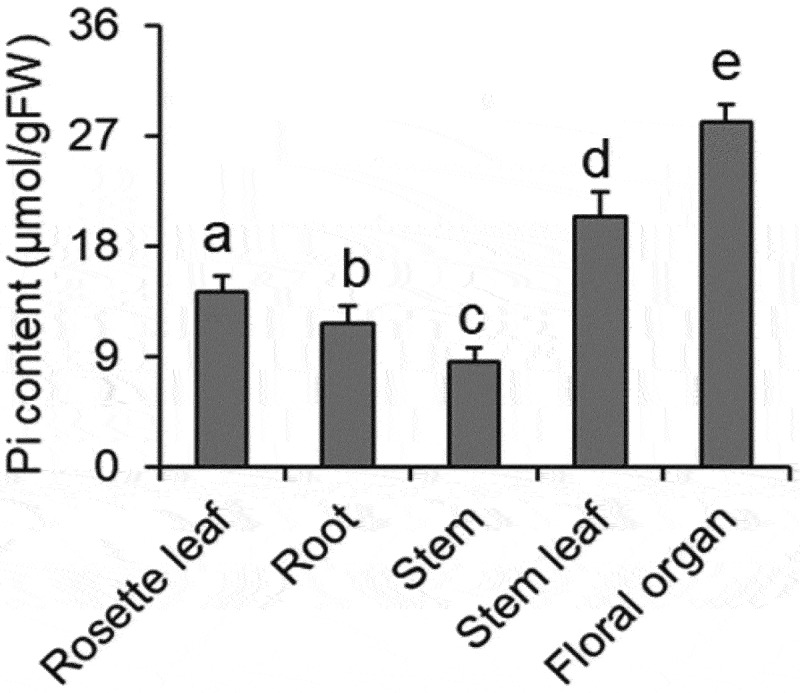
Pi distribution pattern in various tissues. Different tissues including roots, rosette leaf, stem, stem leaf and floral organ were collected from the flowering WT seedlings for Pi content measurements. Different letters above each bar indicate statistically significant differences between various tissues (*P* < 0.05, Tukey’s honestly significant difference test). Error bars indicate ± SD; *n* = 4 biological replicates, each with three technical replicates.

Pi transport in plants is regulated according to nutrient-demanding policies during various development stages. Especially for the flowering，one of the most energy and nutrient consuming development stage, Pi transport should be strictly controlled for reproduction. To detect the Pi distribution pattern in a flowering Arabidopsis plant, we collected different tissues, including roots, rosette leaves, stems, stem leaves and floral organs for Pi content measurements. As shown in Figure 1, the rosette leaves contained more Pi than roots, while the Pi content of stem leaves was higher than that of rosette leaves. The stems contained the lowest Pi, while the Pi content of floral organs was highest among all the tissues.

**Figure 2.**
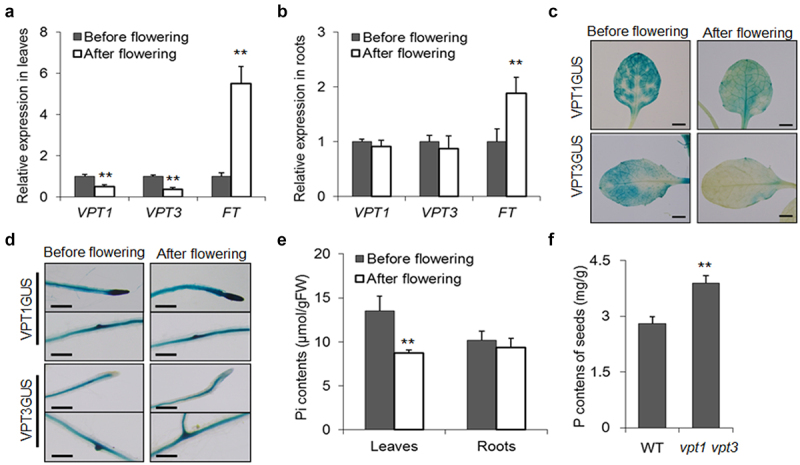
*VPT1* and *VPT3* are transcriptionally regulated for controlling Pi status in the reproductive organ. Expression patterns of *VPT1* and *VPT3* in leaves (a) and roots (b). The leaves and roots of WT seedlings in the indicated development stages were collected for qPCR assays. *FT* gene was used as a flowering indicating maker. Gus stain assay of *VPT1* and *VPT3* in leaves (c) and roots (d), bars represent 2 mm. (e) Pi contents of leaves and roots in different development stages. (f) Seed phosphorus content of various seedlings cultured under Pi sufficient conditions. Stars above each bar indicate statistically significant differences (**P < 0.01, Student’s t test). Error bars indicate ± SD; *n* = 4 biological replicates, each with four technical replicates.

During flowering stage, root-to-flower Pi transport should be precisely regulated to maintain a proper Pi status in the reproductive organ, which contributes to a high quality of production. Our previous study suggested that vacuolar phosphate transporters were very critical for long-distance Pi transport in Arabidopsis, and deletion of two vacuolar influx Pi transporters (VPT1 and VPT3) would result in more Pi delivered into the floral organ and finally affect seed set^11^. To test how *VPT1* and *VPT3* are regulated in different development stages, we conducted qPCR to explore the expression patterns of these two important Pi transporter genes. The data shown in Figure 2a illustrated that before flowering, VPT1 and VPT3 were expressed highly in leaves, whereas a dramatically transcriptional down-regulation occurred when seedlings were full flowering. However, in roots, the transcripts of *VPT1* and *VPT3* are not significantly regulated in different development stages (Figure 2b). The data of GUS staining assay were consistent with the qPCR results (Figure 2c, d). Then, we collected rosette leaves and roots from plants that were in the “before flowering” and the “full flowering” stages separately and measured their Pi contents. The data suggested that Pi contents of roots were not obviously regulated, whereas the Pi content was significantly decreased in “full flowering” leaves than in “before flowering” leaves (Figure 2e). As previous work indicated that loss of function of VPT proteins results in excess of Pi allocated to the floral organs rather than stored in the vacuoles of leaves, we proposed that VPT genes were downregulated for more Pi allocated to the floral organ for meeting the physiological demand of the reproductive organs. During seed maturation, the long-distance transported Pi would be involved in the synthesis of PA that is the major storage form of phosphorus in seeds^12^. Therefore, the more Pi is transported into reproductive organ, the higher PA level in seeds. We conducted the P content measurement of seeds and found that the P level of *vpt1vpt3* double mutant seeds was nearly 30% higher than that of WT (Figure 2f). Thus, downregulation of *VPT1* and *VPT3* in leaves during the flowering stage would enhance P accumulation in seeds, which helps to store enough P source for seed germination.

### Genetic control of VPT1 contributes to reduction of phosphorus contents in seeds

**Figure 3.**
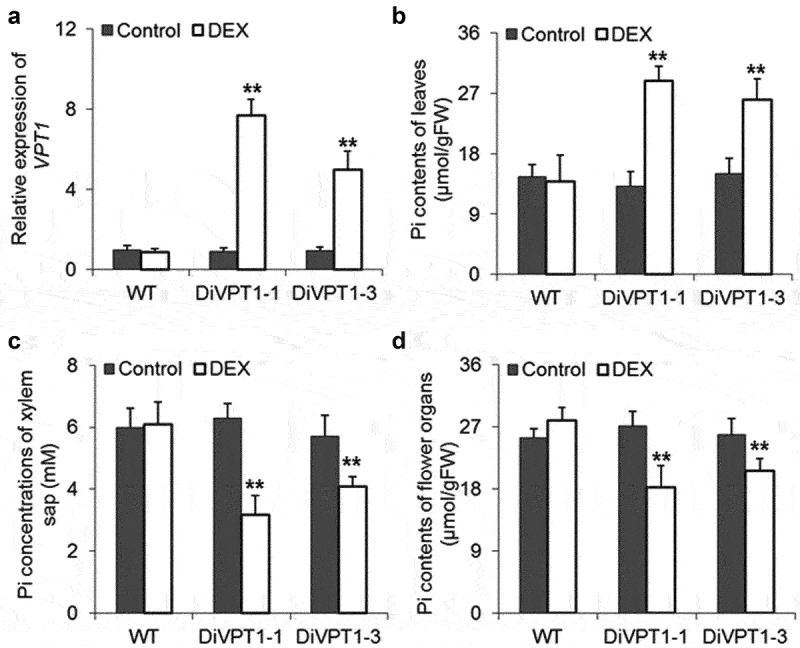
Overexpression of VPT1 in leaves systemically controls Pi long distance transport. (a) The expression level of *VPT1* in the transgenic line was induced by dexamethasone. Wild-type seedlings were used as control. Values were normalized to *UBQ10*. Overexpression of VPT1 in leaves lead to over-accumulation of Pi in leaves (a), lower Pi concentration in xylem sap (b) and decreased Pi contents in floral organs (c). For every figure, Student’s t test, **P < 0.05, error bars indicate ± SD; *n* = 4 biological replicates, each with three technical replicates.

As described above, downregulation of VPT proteins resulted in more Pi allocated to reproductive organs. Therefore, overexpression of VPT1, the major vacuolar Pi influx transporter, may reduce Pi contents in the reproductive organ. To verify this hypothesis, we generated dexamethasone-inducible *VPT1* overexpression lines (DiVPT1). After spraying dexamethasone regent on the surface of DiVPT1 leaves, we found that the expression of VPT1 was significantly upregulated in the leaves (Figure 3a). We then gathered leaves, xylem soup and floral organs for measuring Pi contents. The data shown in Figure 3b,c illustrated that induction of VPT1 dramatically upregulated Pi contents of leaves, while the Pi concentration in xylem soup was significantly downregulated. Meanwhile, the floral organ Pi content of VPT1 inducing seedlings was lower than that of WT and the un-induced seedlings (Figure 3d). Therefore, overexpression of VPT1 in leaves would systemically control the Pi distribution in plants.

**Figure 4.**
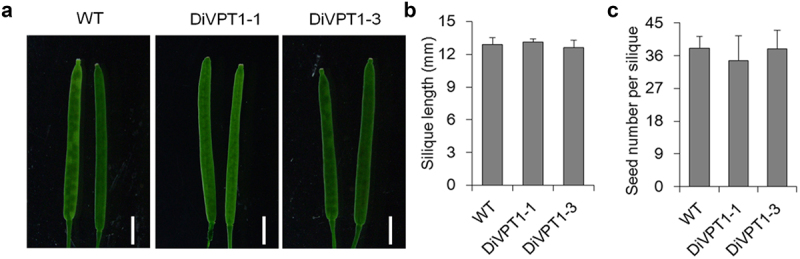
Overexpression of VPT1 in leaves does not affect silique development and seed set. (a) Representative siliques of various seedling lines cultured under sufficient Pi conditions. White bars indicate 2cm. The silique length (b) and the seed number (c) of various seedlings. Student’s t test, data are mean, error bars indicate ± SD; *n* = 10 biological replicates, each with three technical replicates.

**Figure 5.**
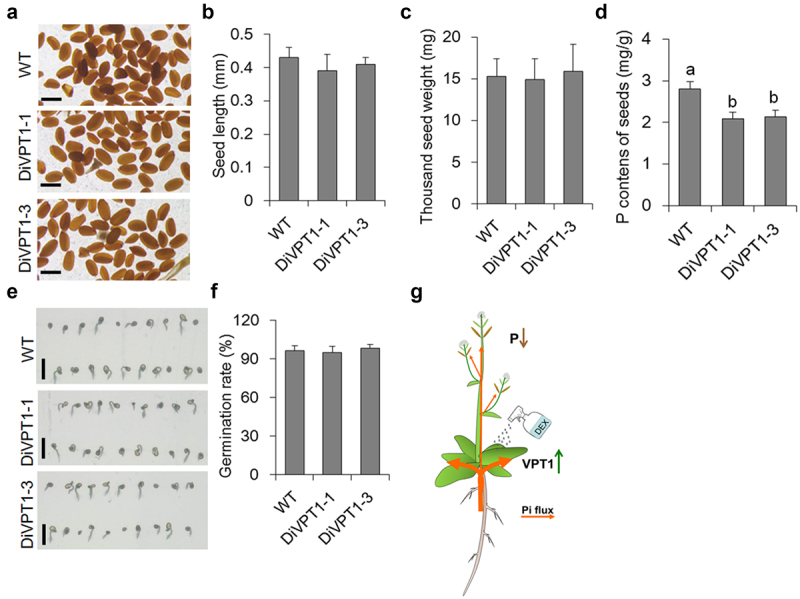
Induction of VPT1 in leaves contributes to lower phosphorus content in seeds. (a) Seeds of various genotypes, bars indicate 0.4 mm. Seed size (b), thousand seed weight (c) and seed phosphorus contents (d) of various seedlings cultured under Pi sufficient conditions. Different letters above each bar indicate statistically significant differences between seedling lines (*P* < 0.05, Tukey’s honestly significant difference test). Error bars indicate ± SD; *n* = 4 biological replicates, each with three technical replicates. (e) Seed germination of various genotypes. Bars, 2mm. (f) Germination rate of various genotypes as shown in (e). (g) Model underlying genetic control of VPT1 reduces phosphorus level in seeds. The green arrow indicates upregulation, while the brown arrow indicates downregulation.

As induction of VPT1 in leaves resulted in less xylem Pi, the P level in seeds may also be downregulated. Then, we cultured WT and DiVPT1 seedling lines for phenotyping. Firstly, we found that overexpressing VPT1 in leaves of DiVPT1 lines did not affect silique development and seed production. The length and seed number of the DiVPT1 siliques were at a comparable level as compared to WT (Figure 4). Moreover, the seed size and the weight of thousand seeds of DiVPT1 lines were also similar with WT (Figure 5a-c). However, the P contents of VPT1-induced DiVPT1 seedlings were more than 20% lower than that of WT (Figure 5d). We then analyzed the germination rates of the various seeds and found that the lower phosphorus containing seeds had a comparable germination rate when compared with WT seeds (Figures 5(e,f)). Thus, overexpression of VPT1 contributes to low P containing seeds without affecting the seed vigor.

In summary, this study explored the strategy of reducing P level in seeds. We firstly tested the Pi distribution pattern in plants during the reproductive stage, and we found that *VPT1* and *VPT3* genes, the major Pi transporters responsible for maintaining systemic Pi homeostasis, were regulated for monitoring Pi distribution among different tissues. The plants tend to suppress VPT1 and VPT3 for stimulating Pi allocation into reproductive organs rather than storage in leaves and thus resulted in high P seeds. Aimed at this case, we successfully downregulated the seed P content by site-specific overexpression of VPT1 with an inducible system (Figure 5g). Recent studies suggested that reducing Pi allocated in grains would result in decreasing PA in seeds and enhanced grain filling for rice production^12,13^, which would contribute to a more environmentally friendly and sustainable agriculture. Therefore, maintaining a low content of Pi in the reproductive organ is important in crops. Based on the data in the study here, we believe that genetically engineering VPT-type proteins in crops such as rice and maize may be a promising strategy to generate low-P containing grains. Moreover, our study strengthens the notion that transporters represent the ideal control points and genetic control of the important nutrient transporters will surely benefit the sustainable agriculture in the future.

## Materials and methods

### Plant materials and growth conditions

Arabidopsis Col-0 seedlings were used in this research. The *vpt1–1* (SAIL_96_H01), T-DNA insertion mutant, was purchased from the Arabidopsis stock center. The hydroponic culture solution was prepared as the recipes in the previous study^14^. For the dexamethasone treatment, 10 µM dexamethasone solutions were sprayed on leaves of the flowering DiVPT1 seedling lines. We treated DiVPT1 seedlings with dexamethasone every 5 d in the early flowering stage till to seed mature stage. *vpt1 vpt3* double mutant used in this study is *vpt1–1 vpt3*^11^. All the seedling lines were grown under a long day cycle (16-h light/8-h dark) at 22°C.

### Vector construction and plant transformation

For the dexamethasone-induced VPT1 vector, the stop codon omitted CDS of VPT1 was amplified from wild type cDNA and cloned into a modified binary vector pCAMBIA-1300 with a dexamethasone-inducing promoter. For the GUS assay vectors, the native promoters of *VPT1* and *VPT3* were fused with GUS and cloned into a modified binary vector pCAMBIA-1300. The primers used for plasmids constructions are listed in Supplementary Table S1. The *Agrobacterium tumefaciens* stain carrying indicated construct (GV3101-pSoup19) was used to transform Arabidopsis Col-0 seedlings.

### GUS staining

The GUS staining was performed as previously described but with small modifications. Briefly, the tissues of stable transgenic seedlings were fixed by incubation in 80% acetone for 15 min and then washed three times with the washing buffer (50 mM NaPO_4_ pH 7.2, 1 mM K_3_Fe[CN]_6_, and 1 mM K_4_Fe[CN]_6_). Then, the plant tissues were submerged in the staining buffer (50 mM NaPO_4_ pH 7.2, 10 mM Na_2_EDTA, 0.1% Triton X-100, 1 mM K_3_Fe[CN]_6_, and 1 mM K_4_Fe[CN]_6_, 2 mM 5-bromo-4-chloro-3-indolyl-β-D-glucuronide) and vacuumed for 20 min. Finally, the tissues with staining buffer were incubated for 8 h in the dark at 37°C. The stained tissues were decolorized with 70% ethanol and photographed.

### RNA isolation and reverse transcription

The plant tissues were immersed in liquid nitrogen and grounded. Total RNA of different samples were extracted by Trizol reagent (Invitrogen). Two-microgram RNA of each sample was used for synthesizing the first-strand cDNA with oligo(dT) primers. qPCR was performed by QuantiFast SYBR Green PCR Kit (Qiagen) on a CFX Connect Real-Time System (Bio-Rad). The specific primer pairs used in qPCR are listed in Table S1.

### Xylem sap collection

The flowering WT and stable transgenic DiVPT1 lines were cultured in the hydroponic solution with sufficient Pi concentration (130 µM). After spraying dexamethasone, the plants were decapitated at the bottom of the stem. The first drop of the xylem sap was discarded, and the subsequent sap was collected every 15 min for 60 min. High humidity (relative humidity is more than 70%) of the plant culture environment is necessary during this experiment. The xylem sap was centrifuged at 12,000 rpm for removing debris, and 20 µl supernatant of each sample was used for Pi concentration measurement or stored at 4°C for later assays.

### Pi content assay

Different tissues of various genotype seedlings grown in the hydroponic culture were collected and washed three times in distilled water. We used 50 mg of each tissue samples or 20 µl xylem sap from various genotype seedlings for Pi content measurement following the ascorbate-molybdate antimony method as previously described^4^.

### Determination of the seed P contents

Phytic acid (PA, or InsP6) is the major form of P in seeds^6–8^, and we therefore measured the seed P content through nitric acid digestion method. Briefly, 20 mg of each seed sample from different genotypes were digested in the liquid nitric acid, so that all the organic phosphorus in seeds were transformed to be inorganic phosphorus (Pi). Then, the digested liquid samples were gathered for Pi measurement through ascorbate-molybdate antimony protocol as previously described.

## Statistical analysis

The data in this study are average values from at least three independent experiments, and the values were subjected to statistical analysis through one-way analysis of variance (ANOVA) followed by Student’s t-test or Tukey’s honestly significant difference test.

## Accession numbers

Sequence data for genes presented in the current study can be found in the Arabidopsis Genome Initiative of GenBank/EMBL database under the following accession numbers: *VPT1* (AT1G63010), *VPT3* (AT4G22990), *UBQ10* (AT4G05320), *FT* (AT1G65480).

## Supplementary Material

Supplemental Material

## References

[1] Luan M, Tang RJ, Tang Y, Tian W, Hou C, Zhao F, Lan W, Luan S. Transport and homeostasis of potassium and phosphate: limiting factors for sustainable crop production. J Exp Bot. 2017;68(12):3091–6. doi:10.1093/jxb/erw444. 27965362

[2] Wang Y, Chen YF, Wu WH. Potassium and phosphorus transport and signaling in plants. J Integr Plant Biol. 2021;63(1):34–52. doi:10.1111/jipb.13053. 33325114

[3] Liu J, Fu S, Yang L, Luan M, Zhao F, Luan S, Lan W. Vacuolar SPX-MFS transporters are essential for phosphate adaptation in plants. Plant Signal Behav. 2016;11(8):e1213474. doi:10.1080/15592324.2016.1213474. 27467463 PMC5022419

[4] Liu J, Yang L, Luan M, Wang Y, Zhang C, Zhang B, Shi J, Zhao FG, Lan W, Luan S. A vacuolar phosphate transporter essential for phosphate homeostasis in Arabidopsis. Proc Natl Acad Sci U S A. 2015;112(47):E6571–8. doi:10.1073/pnas.1514598112. 26554016 PMC4664319

[5] Xu L, Zhao H, Wan R, Liu Y, Xu Z, Tian W, Ruan W, Wang F, Deng M, Wang J, et al. Identification of vacuolar phosphate efflux transporters in land plants. Nat Plants. 2019;5(1):84–94. doi:10.1038/s41477-018-0334-3. 30626920

[6] Karlen DL, Flannery RA, Sadler EJ. Aerial accumulation and partitioning of nutrients by corn. Agron J. 1988;80(2):232–242. doi:10.2134/agronj1988.00021962008000020018x.

[7] Raboy V. Approaches and challenges to engineering seed phytate and total phosphorus. Plant Sci. 2009;177(4):281–296. doi:10.1016/j.plantsci.2009.06.012.

[8] Rose TJ, Liu L, Wissuwa M. Improving phosphorus efficiency in cereal crops: is breeding for reduced grain phosphorus concentration part of the solution? Front Plant Sci. 2013;4:444. doi:10.3389/fpls.2013.00444. 24204376 PMC3817843

[9] Wang F, Rose T, Jeong K, Kretzschmar T, Wissuwa M. The knowns and unknowns of phosphorus loading into grains, and implications for phosphorus efficiency in cropping systems. J Exp Bot. 2016;67(5):1221–1229. doi:10.1093/jxb/erv517. 26662950

[10] Raboy V. Seeds for a better future: ‘low phytate’ grains help to overcome malnutrition and reduce pollution. Trends Plant Sci. 2001;6(10):458–462. doi:10.1016/S1360-1385(01)02104-5. 11590064

[11] Luan M, Zhao F, Han X, Sun G, Yang Y, Liu J, Shi J, Fu A, Lan W, Luan S. Vacuolar phosphate transporters contribute to systemic phosphate homeostasis vital for reproductive development in Arabidopsis. Plant Physiol. 2019;179(2):640–655. doi:10.1104/pp.18.01424. 30552198 PMC6426424

[12] Yamaji N, Takemoto Y, Miyaji T, Mitani-Ueno N, Yoshida KT, Ma JF. Reducing phosphorus accumulation in rice grains with an impaired transporter in the node. Nature. 2017;541(7635):92–95. doi:10.1038/nature20610. 28002408

[13] Ma B, Zhang L, Gao Q, Wang J, Li X, Wang H, Liu Y, Lin H, Liu J, Wang X, et al. A plasma membrane transporter coordinates phosphate reallocation and grain filling in cereals. Nat Genet. 2021;53(6):906–915. doi:10.1038/s41588-021-00855-6. 33927398

[14] Liu TY, Huang TK, Yang SY, Hong YT, Huang SM, Wang FN, Chiang SF, Tsai SY, Lu WC, Chiou TJ. Identification of plant vacuolar transporters mediating phosphate storage. Nat Commun. 2016;7(1):11095. doi:10.1038/ncomms11095. 27029856 PMC4821872

