# Supplementary material for: Genetically controlling VACUOLAR PHOSPHATE TRANSPORTER 1 contributes to low-phosphorus seeds in Arabidopsis
Source: Plant Signal Behav. 2023 Mar 8;18(1):2186641. doi: 10.1080/15592324.2023.2186641 (PMC10012917; doi:10.1080/15592324.2023.2186641)
Supplement: Supplemental Material [file KPSB_A_2186641_SM1456.docx]

**Supplementary table 1. Primers used in this study**

| VPT1 clone primers | VPT1-F: ATGGTGGCTTTTGGGAAATACTTGC  VPT1-R: ATAGAGTGAGTTATAAGTACAACAAGTAGC |
| --- | --- |
| qPCR primers | *VPT1*-q-F: TAACCAATCTACTTTGCCGGGAT  *VPT1*-q-R: CTCTGAATGAAATGCATAGCCATAC  *VPT3*-q-F: GGATCGGCGAGAGCAGTGAA  *VPT3*-q-R: CTTTAACGGTACACAGTCGCTTATAT  *FT*-q-F: AGAGTGGCTGCGGAGGAAGAAG  *FT*-q-R: GCATCATCACCGTTCGTTACTCGTAT  *UBQ10*-q-F: GGCCTTGTATAATCCCTGATGAATAAG  *UBQ10*-q-R: AAGAGATAACAGGAACGGAAACATAGT |
| GUS vector primers | *VPT1-*GUS-F: GGCAGATTATTAAATATTCCACTTTCCAAC  *VPT1-*GUS-R: AGCCACCATCTTTTAATCGCAGAAAG  *VPT3-*GUS-F: GGACCACGTTTCCCATTCATGCA  *VPT3-*GUS-R: GGCTACCATCTTCTACACTGAAAGAAAGT |
